# The role of astigmatism in myopia development, myopia progression and myopia control

**DOI:** 10.1111/opo.70030

**Published:** 2025-10-30

**Authors:** Stephanie Kearney, Rakhee Shah, Natalia Vlasak

**Affiliations:** ^1^ Vision Sciences, Health and Life Sciences Glasgow Caledonian University Glasgow UK; ^2^ Optometry and Visual Sciences City St George's, University of London London UK; ^3^ HOYA Vision Care Amsterdam The Netherlands

**Keywords:** astigmatism, myopia, myopia control

## Abstract

**Aims/Purpose:**

Myopia arises primarily due to excessive axial elongation and is associated with an increased risk of ocular complications and visual impairment, particularly in high myopia. Although astigmatism may have implications on refractive development, the role of astigmatism in myopic eye growth and the effectiveness of myopia control strategies is unclear.

**Methods:**

A scoping review was conducted in March 2025 and updated in August 2025, using MEDLINE and PubMed to explore myopia prevalence and onset, progression and astigmatism (Aim 1) and myopia control and astigmatism (Aim 2). Literature was initially screened by title, abstract and finally by the relevance of the full manuscript.

**Results:**

A total of 1004 studies were identified; 26 duplicates were removed; 911 were excluded following title screening and 20 were excluded following abstract screening. A total of 47 studies were included (Aim 1 = 30, Aim 2 = 17), with most conducted in Asia (*n* = 31). A higher prevalence of astigmatism was associated with myopia. Myopia progression and astigmatism may be related, although there is limited research on the effects of uncorrected astigmatism on the course of myopia. Additionally, there is limited research regarding the influence of astigmatism on the effectiveness of myopia control strategies, with conflicting findings between studies.

**Conclusions:**

Myopia progression and astigmatism may be interdependent. However, a causative role of astigmatism in promoting myopia is unclear. The diverse range of study designs and methodologies impairs the comparability of findings. Prospective research in children of differing ethnicities with a range of astigmatism is required to determine if astigmatism influences the effectiveness of myopia control interventions. This would provide an evidence base to inform clinical decision‐making and management plans for myopic children with astigmatism, particularly in non‐Asian populations where research is limited.

**Key points**

- While many studies report associations, few explored a causative role of astigmatism in myopia. Astigmatism may indicate a greater likelihood for myopia progression and the prevalence of astigmatism increases as myopia progresses.
- Most research reporting the effectiveness of myopia control interventions excluded children with moderate or high astigmatism. There may be an interaction between myopia control interventions and astigmatism, but prospective research in children of differing ethnicities and with a range of astigmatism is required.
- Research into the role of astigmatism, both corrected and uncorrected, concerning the development and progression of myopia and in the effectiveness of myopia control interventions is required. This information is needed to provide an evidence base to inform clinical decision‐making and patient management plans for myopic children with astigmatism.

## INTRODUCTION

Myopia occurs primarily due to an excessive increase in axial length (AL) during childhood. The image is focused in front of the retina, resulting in blurred distance vision, requiring spectacles or contact lenses to correct and facilitate clear vision. Myopia progresses, on average, until the late teenage years.^1^ It increases the risk of secondary ocular complications and visual impairment, particularly in high myopia.^2^ Recently, there has been an increase in the prevalence of myopia across the world,^3^, ^4^ likely due to changes in lifestyle including less time spent outdoors^3^ and increased near work activities. This has been reported in countries including Taiwan, where the prevalence of myopia amongst 7‐ and 12‐year‐olds increased from 5.8% and 36.7%, respectively, in 1983, to 21% and 61% by 2000.^5^ This finding is not confined to Asia, with other countries, including the United States,^6^ also noting an increase in prevalence from 25% to 41.6% for those 12–54 years of age between 1971–1972 and 1999–2004. In the United Kingdom, a similar pattern has also been observed, with the number of Scottish children aged 3.5–5.5 years who failed vision screening due to myopia increasing from <7.8% annually in 2013–2020 to 10.7% by 2021–2022.^7^ In response to this growing health concern, specialised spectacles and contact lenses have been developed and are now available to help slow progression^8^ by reducing the level of myopia accrued by adulthood and, consequently, reducing the risk of ocular complications and visual impairment.

Astigmatism occurs most commonly when parallel rays of light focus at two focal lines perpendicular to one another, instead of at a single focal point, resulting in blurred vision. It can result from variations in the curvature or refractive index of the eye's optical components. Most commonly, differences in corneal curvature along the two principal meridians result in astigmatism.^9^, ^10^ As with myopia, spectacles or contact lenses can be used to correct vision. A recent systematic review and meta‐analysis reported that astigmatism was the most common refractive error amongst adults across the world, with an estimated pooled prevalence of 40%.^11^

Astigmatism tends to be common during infancy and declines during early childhood.^12^ It remains stable during adolescence and early adulthood^9^ before increasing with advancing age.^9^, ^13^, ^14^ Astigmatism can be defined as being with the rule (WTR), where the steeper axis is close to vertical, against the rule (ATR), where the steeper axis is close to horizontal or oblique (where the axes are perpendicular to one another between 30° and 60° or 120° and 150°). Astigmatism is generally WTR during childhood, adolescence and early adult years before becoming increasingly ATR with advancing age.^9^, ^10^, ^14^ Astigmatism may arise from an irregularly shaped corneal surface, primarily the anterior surface, and/or from the crystalline lens. Amongst children,^15^, ^16^ corneal astigmatism is the most significant contributor to total astigmatism.

The exact cause of astigmatism is unclear, but potential causes may include genetics, ethnicity, eyelid pressure and the environment, including excessive screen use and changes to lifestyle such as those reported during the COVID‐19 pandemic, with greater time being spent indoors.^17^, ^18^, ^19^ A recent study has reported that the prevalence and severity of astigmatism increased following COVID‐19 lockdowns.^19^ This has also been the case for myopia, whereby an increase in the prevalence and progression of myopia has been reported.^20^, ^21^, ^22^ This may indicate a shared effect of lifestyle and environment on refractive progression for both astigmatism and myopia.

The role of astigmatism in the myopic eye growth mechanism is unclear. It is proposed that the sensitivity of the focusing mechanism is reduced in myopia,^23^ and therefore chronic blur due to significant astigmatism may degrade further the eye's ability to regulate emmetropisation. This has been evidenced in animal studies where astigmatism has been reported to disrupt normal patterns of eye growth in monkeys and chicks.^24^, ^25^, ^26^ Conversely, it has also been proposed that astigmatism may in fact help facilitate emmetropisation by providing cues to the sign of defocus, which then helps guide normal eye growth.^27^ Regardless of this contradiction, these findings require exploration in humans.

The choroid is a vascular structure located between the retina and sclera. It has been implicated in myopic development such that a thicker choroid may act as a barrier to growth, and that the choroid may be involved in regulating growth factors and biochemicals which may stimulate eye growth.^28^ Myopia has been associated with thinning of the choroid,^29^ and myopia control interventions may result in protective thickening of the choroid,^28^ suggesting that this structure is an important biomarker in myopia development and management. Astigmatism has also been associated with changes in choroidal thickness and vascular density, and this seems to vary depending on the type of astigmatism.^30^, ^31^ Again, this suggests similarities in the processes resulting in astigmatism and myopia.

Myopia control spectacles and contact lenses are widely reported to slow the progression of myopia.^8^ Clinical trials exploring the efficacy of these interventions, including spectacle lenses which are available in a range of prescriptions and can correct considerable levels of astigmatism, tend to exclude children with moderate or high levels of astigmatism.^32^, ^33^, ^34^, ^35^, ^36^ This information is important to determine if children with moderate to high levels of astigmatism are likely to obtain similar treatment effects to those with largely spherical refractive errors, and whether these interventions may influence astigmatism. There is also a lack of evidence as to whether the level of corrected or uncorrected astigmatism may influence the effectiveness of interventions. This may have ramifications for clinical decision‐making and the potential effectiveness of an intervention. This information is required to ensure that evidence‐based decisions and discussions can be held between clinicians, parents and children.

Therefore, astigmatism may affect normal eye growth patterns and astigmatism and myopia may be correlated. This scoping review aimed to explore the association between myopia prevalence and onset, its progression and astigmatism (Aim 1) and examine the association between astigmatism and the effectiveness of myopia control interventions (Aim 2).

## METHODS

Research conducted in human participants and manuscripts published in English were included while letters and commentaries were excluded. Prospective randomised controlled trials, observational, cross‐sectional, experimental, case–control, and retrospective studies were included. Although eligible, no case reports, case series or conference abstracts were included in this review.

Screening was performed independently by author SK. Any queries raised were discussed with author RS. There were no disagreements. Author RS supported the screening process, including the selection of search terms. Searches of PubMed and MEDline were conducted between 23 February and 9 March 2025 and an updated search was performed on 5 August 2025. The search terms relating to the first aim included:

*(myopia) AND (astigmatism OR cylinder OR toric)*

*(axial elongation OR myopia progression) AND (astigmatism OR cylinder OR toric)*

The search terms relating to the second aim included:

*(myopia control OR myopia management) AND (astigmatism OR cylinder OR toric)*

**FIGURE 1:**
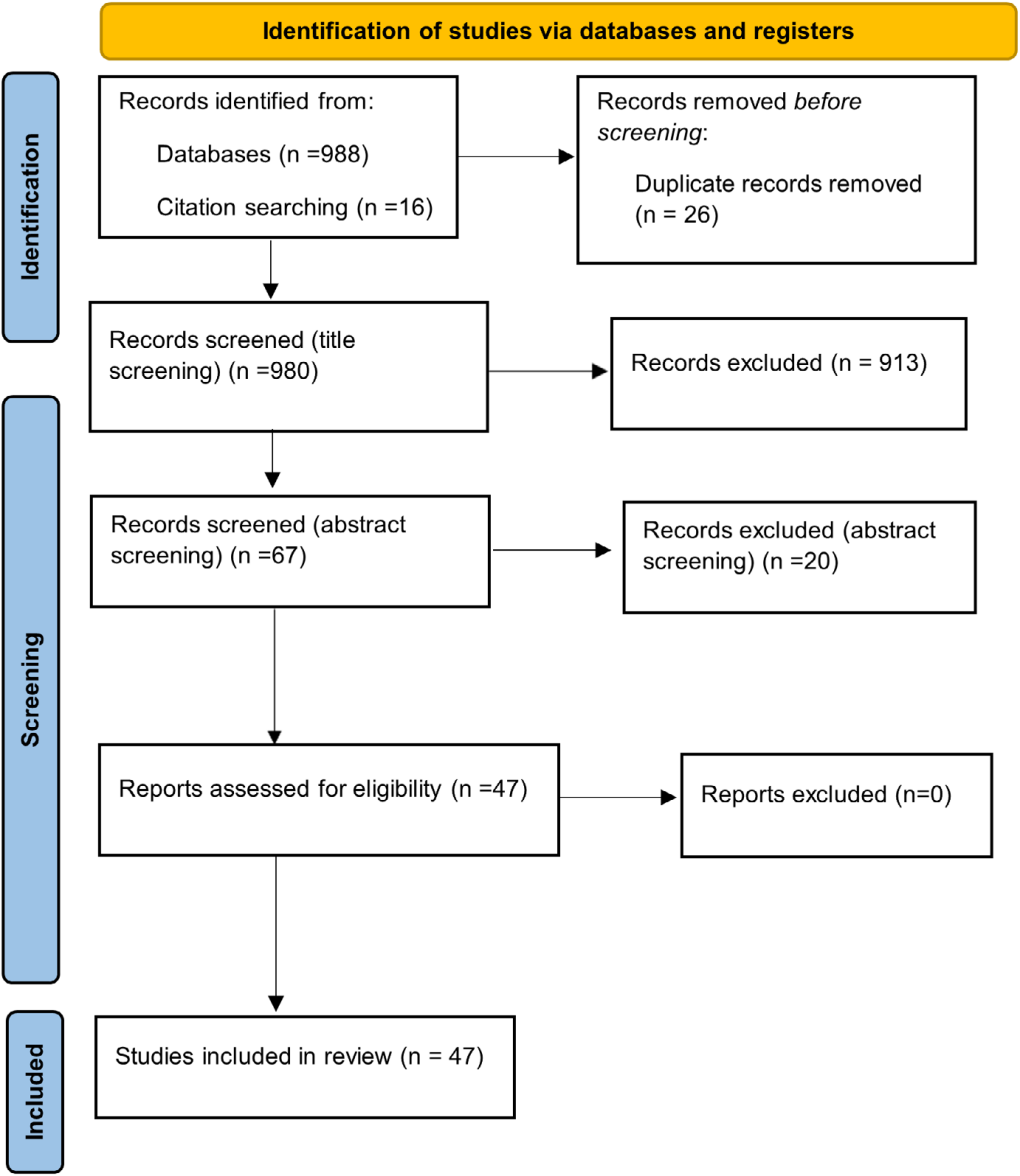
Schematic reporting of the scoping literature review using the Preferred Reporting Items for Systematic reviews and Meta‐Analyses (PRISMA) flow diagram. Manuscripts were initially screened by title and abstract, and finally the full manuscript was evaluated.

Once duplicates were removed, manuscripts were initially screened by title, then by abstract and finally the full manuscript was reviewed in accordance with the inclusion criteria. A total of 1005 manuscripts were initially identified (Figure 1). Following removal of duplicates (*n* = 26), a total of 979 manuscripts were screened by title and 913 were excluded. A total of 68 manuscripts were screened by abstract and 20 were removed. This resulted in a total of 47 manuscripts (30 for Aim 1 and 17 for Aim 2). There were no case reports, case series or conference abstracts. The data extracted included the study design, location, methods, sample size, main findings and limitations.

## RESULTS

A summary of the studies included in the scoping review is included in Table 1. The studies were categorised by study design, age of participants and the region where the study was conducted. The methods used to measure and categorise astigmatism are outlined in Table 2. These varied between studies and included refractive astigmatism (or cylinder), which was determined from the participants' refractive error and/or corneal astigmatism determined from corneal curvature measurements. A summary of the main study outcomes and limitations relevant to Aim 1 (Table 3) and Aim 2 (Table 4) is provided below.

**TABLE 1:** Table summarising study designs including the study type, age of participants and region.

Study design	Region	Study reference	Sample size, respectively
Cross‐sectional = 8	China = 5	37, 38, 39, 40, 41	*n* = 1831, *n* = 7084, *n* = 135 *n* = 99,515, *n* = 4040
	Hong Kong = 1	42	*n* = 418
	Iran = 1	43	*n* = 5528
	Japan = 1	44	*n* = 800 (eyes)
Longitudinal observational = 13	USA = 3	45, 46, 47	*n* = 777, *n* = 383, *n* = 245
	China = 4	48, 49, 50, 51	*n* = 362, *n* = 10,732, *n* = 5961, *n* = 1463
	Finland = 1	52	*n* = 145
	Hong Kong = 2	53, 54	*n* = 382, *n* = 32
	Singapore = 1	55	*n* = 1019
	United Kingdom = 2	56, 57	*n* = 738, *n* = 1053
Case control = 1	China = 1	58	*n* = 50
Cross‐sectional with longitudinal subset = 1	Hong Kong = 1	59	*n* = 108 in the longitudinal aspect *n* = 522 in the cross‐sectional aspect
Retrospective = 14	USA = 5	60, 61, 62, 63, 64	*n* = 16, *n* = 298, *n* = 217, *n* = 102, *n* = 275
	China = 6	65, 66, 67, 68, 69, 70	*n* = 249, *n* = 62, *n* = 1057, *n* = 300, *n* = 69, *n* = 179
	Germany/Hungary = 1	71	*n* = 62
	India = 1	72	*n* = 2683
	United Kingdom = 1	73	*n* = 53 high myopes included in linkage analysis *n* = 90,884 included in cross‐sectional analysis
Nonrandomised experimental study = 3	China = 2	74, 75	*n* = 280, *n* = 400
	Hong Kong = 1	76	*n* = 80
Randomised controlled trial = 7	China = 3	77, 78, 79	*n* = 383, *n* = 119
	Finland = 3	80, 81, 82	*n* = 238, *n* = 238, *n* = 238
	Singapore = 1	83	*n* = 400
Total number of studies by region	China		21
	Hong Kong		5
	Singapore		2
	Japan		1
	Iran		1
	India		1
	Germany/Hungary		1
	Finland		4
	United Kingdom		3
	USA		8
Children only (<16 years) = 40	USA = 7	45, 46, 47, 60, 61, 63, 64	
	China = 18	37, 38, 39, 41, 48, 49, 50, 51, 58, 65, 66, 67, 68, 70, 74, 75, 77, 78, 84	
	Finland = 4	52, 80, 81, 82	
	Germany/Hungary = 1	71	
	Hong Kong = 5	42, 53, 54, 59, 76	
	Singapore = 2	55, 83	
	United Kingdom = 2	56, 57	
	Iran = 1	43	
Children and adolescents (up to 20 years) = 4	China 32	40, 69, 79	
	India = 1	72	
	United Kingdom = 1	73	
Adults (>20 years) = 1	Japan = 1	44	
All ages = 1	USA = 1	62	

**TABLE 2:** Table summarising the measurement and categorisation of astigmatism.

Parameters measured	Method used/equipment	Study reference
Corneal astigmatism	Keratometry (method not specified) = 2	37, 63
	Videokeratometry = 2	39, 44
	Autokeratometry = 3	38, 50, 83
	Zeiss IOLMaster (zeiss.com) = 5	57, 70, 74, 75, 77
	Photokeratoscope = 2	52, 82
	Topography = 6	65, 66, 68, 69, 76, 81
Internal astigmatism	Mathematical calculation = 2	37, 74
Refractive astigmatism (Cylinder)	Non‐cycloplegic autorefraction = 6	38, 40, 42, 49, 53, 58
	Cycloplegic autorefraction = 11	37, 43, 45, 48, 50, 51, 55, 56, 57, 59, 70, 71, 79
	Cycloplegic subjective refraction = 5	62,^a^ 66, 72, 80, 82
	Non‐cycloplegic subjective refraction = 1	74
	Subjective refraction (cycloplegia not specified) = 1	64
	Retinoscopy = 4	46, ^b^, 47, ^c^, 54, ^d^, 60, ^b^
	Cycloplegic retinoscopy = 3	41, 61, 78
	Method of refraction not specified = 3	60, 67, 73
Level of astigmatism included	Up to 1.50DC = 1	83
	With the rule up to 1.50DC = 1	58
	Up to 2.00DC = 7	52, 70, 74, 78, 80, 81, 82
	Up to 2.50DC = 2	63, 77
	Up to 3.00DC = 2	68, 79
	Up to 4.00DC = 1	69
	With the rule 1.25 to 3.50DC = 1	76
	With the rule 1.50 to 3.50DC = 1	66
	Up to 4.00DC = 1	71
	No upper limit reported (population or cohort‐based studies) = 30	37, 38, 39, 40, 41, 42, 43, 44, 45, 46, 47, 48, 49, 50, 51, 53, 54, 55, 56, 57, 58, 59, 60, 61, 62, 64, 65, 67, 72, 73, 75
Definition of myopia	Myopia cycloplegic SER ≤ −0.25D = 1	61
	Myopia cycloplegic SER ≤ −0.50D = 12	37, 40, 48, 52, 54, 55, 57, 70, 71, 72, 76, 78
	Myopia cycloplegic SER ≤ −0.75D = 3	45, 65, 68
	Myopia cycloplegic SER ≤ −1.00D = 6	41, 66, 69, 77, 83
	Myopia non‐cycloplegic SER ≤ −1.00D = 1	53, 79
	Myopia non‐cycloplegic sphere SER ≤ −0.50D = 4	51, 63, 64, 73
	Myopia cycloplegic not specified SER ≤ −0.50D = 2	46, 67
	High myopia cycloplegic SER ≤ −6.00D = 1	40
	High myopia non‐cycloplegic SER ≤ −6.00D = 1	44
	High myopia non‐cycloplegic sphere ≤ −6.00D = 1	73

Abbreviations: DC, dioptres cylinder; SER, spherical equivalent refraction.

^a^ Cycloplegia not used in adults.

^b^ Does not specify if cycloplegia was used or the type of retinoscopy.

^c^ Near‐retinoscopy procedure used in those younger than 3.5 years and non‐cycloplegic distance retinoscopy for children over 3.5 years of age.

^d^ Cycloplegic retinoscopy used in infants. Non‐cycloplegic retinoscopy followed by subjective refraction for refraction at 7–8 years of age.

**TABLE 3:** Table summarising the main study outcomes and limitations relevant to Aim 1.

Aim 1: The association between myopia, its progression and astigmatism			
References	Main study outcomes	Limitations	Sample size
**Positive findings: Myopia progression and astigmatism**			
49	○ Faster axial elongation occurred in both myopic and non‐myopic eyes with ATR or oblique astigmatism in comparison with those with WTR astigmatism. This was not significant in the older cohort ○ In the older cohort only, those with high astigmatism demonstrated faster axial elongation compared to those with low astigmatism	COVID resulted in incomplete data collection for 2 years Non‐cycloplegic refraction Limited sample of high OBL or high ATR	*n* = 10,732
51	○ Those with increasing astigmatism, in comparison with those with reducing or stable astigmatism, demonstrated greater axial elongation and myopia progression. ○ A higher baseline AL (the top quartile compared with the bottom quartile) increased the risk of the onset of astigmatism	Cycloplegia not used	*n* = 1463
56	○ Increasing J_0_ astigmatism was weakly correlated with a more myopic shift in the spherical component of the refractive error in the older cohort only; this was not replicated in the younger cohort (6–7‐year‐old) ○ There was no change reported in J_45_ (oblique astigmatism)	Risk factors for the development or progression of astigmatism were not explored Unequal numbers between the two samples: younger cohort *n* = 399, older cohort *n* = 669	*n* = 738
59	○ Greater levels of astigmatism were associated with an increased myopic shift and greater axial elongation ○ The type of astigmatism was not associated with SER or AL change ○ Children with increased astigmatism had a more myopic SER, longer AL and a myopic shift in SER and axial elongation	Fewer children in the longitudinal aspect of the study (*n* = 108) in comparison with the cross‐sectional study (*n* = 522). However, characteristics of the children were similar	*n* = 108 in the longitudinal aspect *n* = 522 in the cross‐sectional aspect
61	○ Myopia progression occurred in those with equal to or greater than 1.00DC ○ In those with greater than or equal to 3.00DC, myopia tended to continue to progress until 8 years of age ○ Astigmatism of greater than or equal to 1.00D, particularly oblique astigmatism, was associated with higher levels of myopia	Retinoscopy used Hospital‐based population with a young age of myopia onset	*n* = 298
72	○ Both moderate astigmatism of <0.75 to ≥−2.00DC and high astigmatism (<−2.00DC) increased the risk of myopia progression	Myopia progression was determined using previous spectacle prescription from 1 year ago, compared with cycloplegic subjective refraction	*n* = 2683
**Positive findings: Myopia and astigmatism**			
38	○ A greater level of myopia increased the risk of refractive but not corneal astigmatism	Cycloplegia not used	*n* = 7084
40	○ The amount of myopia increased with increasing astigmatism ○ WTR astigmatism was the most common axis of astigmatism amongst myopes	Cycloplegia not used	*n* = 99,515
41	○ Children with myopia or hyperopia had increased risk of astigmatism ○ A more myopic SER was significantly associated with greater astigmatism	Participants were recruited from low‐income families and those with vision disorders. The sample may not be representative of a more general population	*n* = 4040
42	○ Astigmatic children were more myopic than non‐astigmatic children ○ After accounting for age, gender and parental myopia, myopic eye growth and astigmatism were not strongly related	Cycloplegia not used Unclear how the three primary schools were selected	*n* = 418
43	○ The amount of astigmatism increased with the amount of myopia	The ethnic characteristics of the population were not reported	*n* = 5528
44	○ Highly myopic adults had greater WTR astigmatism than those without high myopia ○ The change from WTR to ATR astigmatism occurred at a later age in those with high myopia compared with those who did not have high myopia. However, the magnitude of change in ATR did not differ between groups	Study confined to adults Study comparisons confined to high myopia	*n* = 800 eyes
46	○ There was a higher number of children with myopia and ATR astigmatism in comparison with those with emmetropia or hyperopia ○ Myopes were more likely to have astigmatism ○ Children with ATR astigmatism tended to become myopic in later years	Not specified if cycloplegia used Retinoscopy used to evaluate longitudinal changes in refractive error	*n* = 383
47	○ SER became less positive both in the first year and after 10 years of age in children with higher amounts of cylinder in infancy	Variable follow‐up of between 6 and 23 years	*n* = 245
50	○ Greater progression of astigmatism was independently associated with a longer axial length at baseline	Sample limited to children aged 7–11 years	*n* = 5961
53	○ Higher prevalence of myopia was accompanied by a higher prevalence of astigmatism	Two schools sampled; may not be fully representative of the population Non‐cycloplegic refraction	*n* = 382
54	○ Astigmatism in infancy was greater for those who were myopic by 7–8 years of age	Cycloplegic retinoscopy in infancy was compared with subjective refraction when aged 7‐8 years old	*n* = 32
55	○ Children with myopia had a higher incidence of astigmatism than those without myopia ○ The 3‐year incidence rate of astigmatism (at least 1D) was greatest in those with myopia ○ Greater J_0_ and J_45_ progression was associated with the presence of myopia at baseline	Non‐random sampling of schools	*n* = 1019
62	○ There was a significant association between the presence of astigmatism and a younger age of myopia onset ○ The level of SER was negatively correlated with cylinder power but was not a risk factor for astigmatism ○ There was no correlation between the type of astigmatism (WTR, ATR or oblique) and the level of myopia	Study population confined to those with a family history of non‐syndromic high myopia	*n* = 217
**Negative findings: Myopia progression and astigmatism**			
63	○ No significant correlation between corneal power and myopia progression	Subjective refraction used	*n* = 102
60	○ There was an increase in myopia when a child changed from mixed astigmatism to simple myopic or compound myopic astigmatism ○ There was little change in refractive error with mixed astigmatism even in the presence of myopia ○ The rate of myopia progression did not vary by WTR, ATR or no astigmatism in myopic children	Small retrospective clinic dataset (*n* = 16)	*n* = 16
64	○ Rate of myopia progression did not vary by WTR, zero or ATR astigmatism	Non‐cycloplegic subjective refraction used	*n* = 275
73	○ Refractive and corneal astigmatism increased with both myopic and hyperopic refractive errors ○ The change in proportion from WTR to ATR with age was similar across all refractive groups ○ WTR astigmatism was most common amongst high myopes	To avoid the effects of age on astigmatism, the sample was confined to those aged 21–30 years	*n* = 53, high myopes included in linkage analysis *n* = 90,884 included in cross‐sectional analysis
80	○ Myopia progression was not correlated with the level of astigmatism at baseline	Subjective refraction used. Only myopia up to 3.00D included	*n* = 238
82	○ There was an association between higher astigmatism and less myopia in those with ATR ○ There were no significant correlations between the level of baseline astigmatism and myopia progression over a 3‐year period	Subjective refraction used. Only myopia up to 3.00D included	*n* = 238
**Negative findings: Myopia and astigmatism**			
37	○ Refractive and corneal astigmatism increased with both myopic and hyperopic refractive errors	School based rather than population based	*n* = 1831
45	○ Refractive error change did not differ significantly with the magnitude of baseline astigmatism	Study confined to native Americans	*n* = 777
48	○ Astigmatism was positively associated with baseline SER but not with AL. ○ At the beginning of the study, those with clinically significant astigmatism (more than 1.00DC) also had a more myopic SER. However, by the end of the study, the opposite was found in that those with non‐clinically significant astigmatism had a greater myopic shift in SER ○ There was no significant difference in SER or AL change when comparing those with an increase in astigmatism versus those with stable or decreasing astigmatism ○ There was no significant correlation between baseline astigmatism and change in SER or AL	Children from one primary school recruited, may not be representative of the population as a whole	*n* = 362
52	○ There was no association between the change in corneal apex position, relative to the visual axis and AL	AL measured using a‐scan ultrasonography	*n* = 145
57	○ The prevalence of astigmatism increased with both myopia and hyperopia, and was not specific to myopia	Low prevalence of myopia in the younger cohort (Aged 6–7 years, [2.0% CI, 0.4–3.6])	*n* = 1053
81	○ No significant relationship was found between anisometropia of astigmatism and the amount of SER	Sample confined to those with anisometropia of spherical equivalent or anisometropia of astigmatism	*n* = 238

Abbreviations: AL, axial length; ATR, against the rule; CI, confidence interval.; D, dioptre; DC, dioptres of cylinder; OBL, oblique; SER, spherical equivalent refraction; WTR, with the rule.

**TABLE 4:** Table summarising the main study outcomes and limitations relevant to Aim 2.

Aim 2: Astigmatism and myopia control interventions			
References	Main findings	Limitations	Sample size
**Positive findings**			
39	○ There were small decreases in corneal power (<0.25D) in myopic eyes in comparison with hyperopic eyes following the use of atropine 1% twice a day for 5 days	Findings are too small to be considered clinically meaningful. Short follow‐up time	*n* = 135
78	○ There was a statistically significant increase in cylindrical refractive error in the 0.01% atropine group in comparison with the control group	One year follow‐up affected by COVID with 14 in the treatment group and 13 in the control group lost to follow‐up Single hospital‐based study Findings are too small to be considered clinically meaningful	*n* = 119
83	○ There was a statistically significant increase in corneal (J_0_) astigmatism in the 1% atropine group in comparison with the control group	Findings are too small to be considered clinically meaningful	*n* = 400
66	○ The rate of axial elongation was not associated with initial corneal astigmatism ○ Axial elongation was significantly less in the toric orthokeratology group than in the spherical orthokeratology group	Data confined to 1‐year follow‐up The mean difference is small and may be within the variability of the instrumentation used	*n* = 62
67	○ An increase in astigmatism occurred which was greatest in the HAL group in comparison with the peripheral modification lenses and control groups. The myopia control effect was greatest in the HAL group	Differences between groups are small and unlikely to be clinically meaningful The method of refraction used was not described	*n* = 1057
71	○ Those with astigmatism demonstrated greater axial elongation while wearing DIMS lenses than those with a spherical refractive error. This finding was not observed in those wearing single vision lenses	Participants recruited to the intervention group demonstrated at least 0.50D of myopia progression prior to recruitment. This inclusion criteria was not applied to the virtual control group	*n* = 62
69	○ Corneal curvature was identified as a significant predictor of axial elongation in a multivariate analysis model and significantly negatively correlated with axial elongation in those wearing multifocal rigid gas permeable lenses	Retrospective study design	*n* = 69
70	○ A more myopic change in SER was associated with a greater change in astigmatism (change in astigmatism defined as more than 0.25D) in those wearing DIMS lenses ○ This relationship was dose dependent ○ Change in axial length was not associated with change in astigmatism ○ Baseline corneal astigmatism was associated with change in astigmatism	Low threshold for astigmatism change (0.25D) No control group	*n* = 179
**Negative findings**			
58	○ No significant difference in axial length changes between those wearing toric versus spherical orthokeratology lenses	Small case control study (*n* = 25) Due to the retrospective nature of the study, there was no control group	*n* = 50
65	○ No correlation between baseline corneal power or astigmatism with axial elongation	Due to the retrospective nature of the study, there was no control group	*n* = 249
74	○ There was no significant change to corneal curvature or corneal power following the instillation of 0.01% or 0.02% atropine over a 2‐year period	Children could select whether they used atropine (or not), which may increase the risk of bias	*n* = 280
75	○ There was no significant increase in corneal astigmatism (including J_0_ and J_45_) following the use of atropine ○ No significant differences in the change in corneal astigmatism (including J_0_ and J_45_) between the atropine treatment groups	Children could select whether they used atropine which may increase the risk of bias	*n* = 400
76	○ No significant correlation between axial elongation and baseline astigmatism in toric orthokeratology or control (single vision spectacles)	Non‐randomised, parents could select the treatment group their child joined	*n* = 80
77	○ Corneal astigmatism remained stable after 1 year, with no difference between children using 0.05% atropine in comparison with 0.025% and 0.01% atropine	Data confined to 1‐year follow‐up	*n* = 383
68	○ Corneal astigmatism was not associated with axial elongation in myopic children wearing orthokeratology lenses over a 1‐year period	Retrospective data, confined to 1‐year follow‐up	*n* = 300

Abbreviations: DIMS, defocus incorporated multiple segments; HAL, highly aspheric lenslets; SER, spherical equivalent refraction.

### Aim 1: Positive findings relating to myopia progression and astigmatism

Fan et al.^59^ conducted a 5‐year longitudinal study in Hong Kong children with a broad range of refractive errors (−4.00 to +5.00DS; *n* = 108). Greater levels of astigmatism were associated with a larger myopic shift (*r* = 0.31) and more axial elongation (*r* = −0.28). The type of astigmatism was not associated with the spherical equivalent refraction (SER) or AL change. In comparison with children with stable or decreasing astigmatism, those with increased astigmatism had a more myopic SER (−1.19D (1.72D) vs. 0.27D (1.40D)), longer AL (24.10 mm (0.87 mm) vs. 23.48 mm (0.89 mm)), larger myopic shift in SER (−2.25D (1.43D) vs. −0.72D (1.08D)) and greater axial elongation (2.07 mm (0.70 mm) vs. 1.55 mm (0.80 mm)) over the 5‐year period. These findings suggest that an increase in astigmatism may result in an increase in myopic eye growth. It is important to note these results relate to the use of single vision spectacle lenses, as myopia control interventions were not widely used at the time of the publication.

This finding has also been replicated in a Chinese population (*n* = 1463) over a 2‐year period; those with increasing astigmatism (compared with reducing or stable astigmatism) demonstrated greater axial elongation (0.68 mm vs. 0.56 mm vs. 0.53 mm, respectively) and myopia progression (using non cycloplegic autorefraction) (−0.86D, −0.31D and −0.39D, respectively).^51^ Additionally, a higher baseline AL (the top compared with the bottom quartile) increased the risk for the onset of astigmatism (Odds Ratio [OR] = 5.19 (95% CI 2.72–9.90)).^3^

In further support of this argument, Gong et al.^50^ (*n* = 5961) reported that greater astigmatic progression was independently associated with a longer AL at baseline (AL < 23.5 mm = −0.09 ± 0.35DC, AL ≥ 23.5 and < 24.5 mm = −0.15 ± 0.39DC and AL ≥24.5 mm = −0.29 ± 0.44D). The authors suggested that individuals with a longer AL might have less stable astigmatism. However, the effects were small.

Faster axial elongation in both myopic and non‐myopic eyes has been reported in Chinese children (*n* = 7880) with ATR (0.42 mm/year) or oblique astigmatism (0.37 mm/year), compared with WTR (0.29 mm/year) astigmatism over an average follow‐up period of 2.74 (0.98) years.^49^ However, this finding was not significant in the older cohort (*n* = 2852, mean baseline age 12.73 (1.09) years). Although this suggests that astigmatism is associated with both myopia and hyperopia in the older cohort only, those with high astigmatism (≤−1.50D) demonstrated faster axial elongation (0.43 mm/year) compared with those with low astigmatism (>−1.50D to ≤−0.50D) (0.32 mm/year), suggesting an interaction between myopia and astigmatism. A bidirectional trend was found whereby those with WTR astigmatism and compound myopia (both principal meridians ≤−0.75D) demonstrated greater axial elongation (0.25 mm/year) than those with WTR and compound hyperopia (both principal meridians >0.50D) (0.10 mm/year) in the cohort overall, further supporting an interaction between myopia and astigmatism. It should be considered that this study was somewhat limited in its follow‐up with COVID resulting in incomplete data collection for 2 years.

A further Chinese study in a large population of children and young adults aged 6–18 years (*n* = 99,515), also found that the level of myopia increased with greater astigmatism ($X_{\text{trend}}^{2}$ = 2603.927, *p* < 0.05), supporting a relationship between the two conditions.^40^ Additionally, WTR was the most common type of astigmatism amongst those with both low (SER ≤ −0.50D) (53.9% (95% CI 53.5–54.3)) and high myopia (SER ≤ −6.00D) (5.7% (95% CI 5.5–5.9%)).

Huang et al.^41^ reported that in young Chinese children (aged 3–6 years, *n* = 4040) as part of the Vision in Preschoolers study, those with myopia or hyperopia had increased risk of astigmatism with an OR of 4.50 (95% CI: 3.00–6.76) for myopia and 1.55 (95% CI: 1.29–1.86) for hyperopia. Additionally, a more myopic SER was a risk factor for greater astigmatism, with mean astigmatism of +2.74D within the myopic group compared to +2.15D and +2.28D for hyperopes and emmetropic astigmats, respectively.

In young adults aged 18–20 years (*n* = 2683), both moderate astigmatism (<−0.75 to ≥−2.00DC) and high astigmatism (<−2.00DC) increased the risk of myopia progression (OR 2.68 (95% CI 1.49–4.83) and OR 6.60 (95% CI 3.40–12.81), respectively).^72^ This suggests that astigmatism may influence myopic eye growth. However, this was a retrospective study of young adults, which may not be applicable to younger children, particularly those still undergoing emmetropisation. Additionally, myopia progression was determined using the spectacle prescription from 1 year previously and compared with cycloplegic subjective refraction, which may have created variability in the results due to the different methods of refraction used.

Most of the literature that has explored this topic is based on children from East Asia. A study in the USA explored retrospective clinic data from a small sample of 16 children.^60^ They reported an increase in myopia (−0.60DS) when the refractive error changed from mixed to simple myopic or compound myopic astigmatism. The small dataset and retrospective nature of the data may limit the applicability of these findings. Furthermore, the study reported little change in refractive error with mixed astigmatism, even in the presence of myopia.

Another study conducted in the USA (*n* = 245) reported that SER became less positive both in the first year and after 10 years of age in children with higher levels of astigmatism (>1.00DC) in infancy.^47^ Despite statistical significance, the correlation between the change in cylinder and change in SER was low (*r* = −0.20 for those aged 4 months to 2 years and *r* = −0.43 in those aged 6–12 years). This was a longitudinal study with variable follow‐up of between 6 and 23 years (*n* = 245, mean age at last refraction 12.7 years).

In Northern Irish children (*n* = 738), increasing J_0_ astigmatism was correlated significantly with a more myopic shift in the spherical component of the refractive error in the older cohort only (12–13‐year olds; Spearman's ρ = −0.31, *p* = 0.006); this was not replicated in the younger cohort (6–7‐year old) (Spearman's ρ = −0.08, *p* = 0.20).^56^ There was no significant change reported for J_45_ (oblique astigmatism). However, the Spearman's rho value indicated a weak correlation. Unequal sample sizes may also have influenced the results, with greater statistical power in the larger older cohort (*n* = 669) than in the younger cohort (*n* = 399). Additionally, a previous study by the same research group^57^ (*n* = 1053) reported that the prevalence of astigmatism increased with both myopia and hyperopia and was not specific to myopia (6–7‐year‐old children, rho = 0.23; 12–13‐year‐old children, rho = 0.32).

Many studies have explored the effects of corrected astigmatism. However, the retrospective investigation by Fulton et al.^61^ reported that in American children aged ≤3 years (*n* = 298) and not wearing a refractive correction, myopia progression occurred in those with ≥1.00DC. The level of cylinder was positively related to the level of myopia in that a greater level of cylinder was significantly correlated with higher levels of myopia (*r* = 0.47, *p* < 0.01), particularly for those with oblique astigmatism. Additionally, in those with ≥3.00DC, myopia tended to continue to progress until 8 years of age. The authors suggested that uncorrected astigmatism during the period of visual immaturity influences the course of myopia. However, due to the retrospective nature of this study, a causative role of astigmatism in promoting myopic growth cannot be determined. Additionally, these findings may not be directly applicable to ‘school‐aged’ myopia which occurs at a later age and may differ in trajectory from congenital myopia or that occurring at a much younger age.

### Aim 1: Negative findings relating to myopia progression and astigmatism

The lack of a significant association between myopia progression and astigmatism has also been reported. For example, Pärssinen et al.^80^ in Finland (*n* = 238) observed that in the cohort overall, myopia progression was not correlated significantly with the level of astigmatism at baseline (*r* = −0.07, *p* = 0.16). Another publication by the same study group (*n* = 238)^82^ noted that while there was a significant association between higher astigmatism and less myopia in those with ATR (*r* = −0.49, *p* < 0.001), there were no significant correlations between the level of baseline astigmatism and myopia progression over a 3‐year period.^82^ The group also reported that change in corneal apex position over a 3‐year period was not associated with AL (*n* = 145).^52^

A retrospective study in the United States analysed patient records and reported that the rate of myopia progression did not vary by WTR, ATR or no astigmatism in myopic children (*n* = 275).^64^ In an earlier study (*n* = 102), these authors also found no significant correlation between corneal power and myopia progression when analysing retrospective patient records; however, data on AL were not captured.^63^

Refractive error change did not differ significantly with the magnitude of baseline astigmatism in North American children (*n* = 777).^45^ However, the authors reported that when outliers were removed, there was a relationship between greater baseline refractive astigmatism and an increase in myopia progression per year. Nevertheless, the removal of outliers from datasets without due cause may misrepresent the natural distribution of the findings.

The prospective cohort study (*n* = 362) by Chan et al.^48^ reported that at baseline and after 1 year of follow up, astigmatism was positively associated with baseline SER but not with AL (*r* = 0.38, *p* = 0.002). At the beginning of the study, those with clinically significant astigmatism (>1.00DC) also had a more myopic SER. However, by the end of the study, the opposite was found, in that those with non‐clinically significant astigmatism had a greater myopic shift in SER. There was also no significant difference in SER or AL change when comparing those with an increase in astigmatism versus the subjects with stable or decreasing astigmatism. When myopic progression was analysed, there was no significant correlation between baseline astigmatism and the change in SER or AL.

### Aim 1: Positive findings relating to myopia and astigmatism

The longitudinal Singapore Cohort Study of the Risk Factors for Myopia (SCORM)^55^ reported that at baseline, children (*n* = 1019) with myopia had a higher incidence of astigmatism than those without myopia. After controlling for age, gender and ethnicity, the 3‐year incidence of astigmatism (at least 1D) was 4.5% (95% CI: 2.7–6.4) and 29.2% (95% CI: 22.7–35.6%) for those without and with myopia, respectively. Additionally, greater J_0_ and J_45_ progression was also associated with the presence of myopia at baseline.

Wong et al.^42^ explored differences in the level of myopia by the amount of astigmatism (astigmatism >0.75DC) in a cross‐sectional study in Hong Kong children (*n* = 418). The authors reported that astigmatic children were, on average, more myopic by 0.50D and 0.27 mm than non‐astigmatic children. However, after accounting for age, gender and parental myopia, and including astigmatism as an outcome, there was only a modest increase in cylindrical power of 0.18D, as well as 0.10D of J_0_ astigmatism for each 1 mm increase in AL, which suggests that myopic eye growth and astigmatism were not strongly related.

A higher prevalence of both myopia and astigmatism has also been reported in children in two schools in Hong Kong (prevalence of myopia 37.5% vs. 12.8% and prevalence of astigmatism, 25.0% vs. 7.2% for the local [*n* = 159] and international school [*n* = 233], respectively).^53^ However, the generalisability of these findings to the population as a whole may be limited due to insufficient representation of schools. Additionally, these findings were observations that were not analysed statistically. Another study from Hong Kong^54^ (*n* = 32) also reported that astigmatism in infancy was greater for those who were myopic by 7–8 years of age (1.56DC, SD 1.08DC), compared with those who were not myopic (0.75DC, SD 0.74DC). However, as cycloplegic retinoscopy was used in infancy and results compared with subjective refraction at 7–8 years of age, differences in the methods of refraction may impede comparability of results between ages.

Interestingly, a cross‐sectional study in Chinese children (*n* = 7084) reported that a greater level of myopia increased the risk of refractive astigmatism as an outcome, but this was not the case of corneal astigmatism (OR 0.96 95% CI 0.92–0.99).^38^ These conflicting findings may either be due to the use of non‐cycloplegic refraction resulting in greater variability, or it may suggest that optical components other than the cornea may have influenced these results.

Again, much of the research that has explored this topic was carried out on Asian populations. However, Twelker et al.^45^ examined Native American children and young adults (aged 3–18 years, *n* = 777), and reported that those with high astigmatism (>3.00D) were more likely to be myopic by 18 years of age. However, refractive error change did not differ significantly based on the magnitude of baseline astigmatism.

Another study of American children (*n* = 383, aged 5–6 years at baseline) reported a higher number with myopia and ATR astigmatism (*n* = 36) compared with emmetropes (*n* = 19) or hyperopes (*n* = 16).^46^ Additionally, the myopes were more likely to have astigmatism. Children with ATR astigmatism >0.12D tended to become myopic in later years. However, this value is too small to be considered clinically meaningful.

A longitudinal study of American children (*n* = 245, mean age 12.7 years, 5 years of age at baseline) reported that ATR astigmatism was associated with a greater level of myopia after 7 years of age.^47^ However, this study used non‐cycloplegic distance retinoscopy, which may have overestimated the level of myopia.

In children in Iran (*n* = 5528), the level of astigmatism increased with the magnitude of myopia, whereby the lowest amount of astigmatism (0.35D) occurred in those with a spherical refractive error of +1.00D, increasing to around 2.4D of astigmatism in those with myopia >4.50D.^43^

There have also been a small number of studies in adults. For Japanese adults over 40 years of age,^44^ those with high myopia (*n* = 800 eyes) had greater WTR astigmatism than those without high myopia (*n* = 800). Also, the change from WTR to ATR astigmatism occurred at a later age in those with high myopia. However, the magnitude of change in ATR did not differ between the groups. Although these findings were obtained in adults, they suggest that the relationship between myopia and astigmatism may persist throughout adulthood.

In individuals with a family history of non‐syndromic high myopia (*n* = 217, mean age 37.1 years), there was a significant association between the presence of astigmatism and the age of myopia onset, such that a greater number of eyes were astigmatic (*n* = 75) in participants with myopia onset earlier than 6 years of age, in comparison with an age of onset >6 years (*n* = 46).^62^ Additionally, the level of SER was correlated with cylinder power (*r* = −0.34, *p* < 0.001) but was not a risk factor for astigmatism. However, there was no significant correlation between the type of astigmatism (WTR, ATR or oblique) and the level of myopia. As this study was conducted in a specific population, it is unclear if the findings are applicable to a more general population.

### Aim 1: Negative findings relating to myopia and astigmatism

A cross‐sectional study in Chinese children (*n* = 1831) reported that refractive and corneal astigmatism increased with both myopic and hyperopic refractive errors, although this association was not specific to myopia.^37^ This is in agreement with Farbrother et al.^73^ who observed that in young adults (21–30 years, *n* = 10,096), greater levels of astigmatism were associated with higher levels of refractive error in both myopia and hyperopia. Furthermore, the change from WTR to ATR with age was similar across groups and did not vary with the presence of myopia. However, it was noted that WTR astigmatism was most common amongst high myopes (*n* = 53).^73^

In contrast, a study of Chinese children (*n* = 535) reported no significant differences in the change in AL or SER between those with WTR, ATR or oblique astigmatism.^48^ The authors also reported no significant difference in SER or AL change when comparing those exhibiting an increase in astigmatism versus individuals with stable or reducing astigmatism. Research in an American population also confirmed no significant correlation between the type of astigmatism (WTR, ATR or oblique) and the level of myopia^60^ in a retrospective analysis of patient records across five optometry practices.

Pärssinen and Kauppinen^81^ explored the relationship between anisometropia and astigmatism in myopic children (*n* = 238). They reported no significant correlation between aniso‐astigmatism and the level of refractive error. However, as this investigation focused particularly on anisometropia, its comparability with others discussed in the scoping review may be limited.

### Aim 2: Positive findings relating to myopia control and astigmatism

A recent novel study explored whether the level of astigmatism at baseline influenced the rate of myopia progression in children wearing Defocus Incorporated Multiple Segments (DIMS, hoyavision.com) spectacle lenses (*n* = 62).^71^ This retrospective observational study in European children (*n* = 62) reported that 41% of participants in the progressing group were astigmatic (at least 0.50DC). Mean SER and AL change differed significantly between those who were astigmatic versus those without astigmatism (SER: −0.49 ± 0.07D vs. −0.26 ± 0.07D and AL: 0.20 ± 0.05 mm vs. 0.12 ± 0.04 mm, respectively, at 12‐month follow‐up). The difference in SER change was due to a change in spherical rather than astigmatic power. However, when the authors explored whether myopia progression was associated with astigmatism in the virtual control group, which included German children wearing single vision spectacles (LIFE child study),^85^ there was no significant difference in axial elongation between those with and without astigmatism over the 12 month trial (0.12 ± 0.02 mm vs. 0.11 ± 0.01 mm, respectively). There was a difference between the cohorts in that Domsa et al.^71^ recruited children demonstrating at least 0.50D of myopia progression. However, the German population^85^ may be more representative of the general population with a much larger sample size of 1965 participants. Nevertheless, this finding suggests that astigmatism may influence the efficacy of the DIMS spectacle lens. It was also suggested that the DIMS spectacle lens may directly influence the relationship between astigmatism and axial elongation.

Another recent study^67^ of Chinese children (*n* = 1057) explored records from a hospital clinic retrospectively. The investigation explored those wearing highly aspherical lenslets (HAL, Stellest, essilorluxottica.com), peripheral modification lenses (Myovision, zeiss.com) and a control group wearing either single vision spectacles or no correction. Interestingly, the number of children with an annual change in cylinder ≥0.50D was 23.6% (86/365) in the HAL group, 17.1% (56/327) in the peripheral modification group and 16.2% (59/365) in the control group. Additionally, as the rate of myopia progression slowed with the intervention, the level of change in cylinder per year increased, being greatest with the HAL lenses: −0.15 ± 0.33D change/year versus −0.09 ± 0.27D and −0.05 ± 0.33D in the peripheral modification and control groups, respectively. Although these differences were small, the greater cylindrical effect within the HAL group may be due to the larger myopia control effect of these lenses (change in SER/year: −0.23 ± 0.41D) in comparison with the peripheral modification (−0.63 ± 0.43D) and control groups (−0.71 ± 0.52D). The authors also reported no significant correlation between the baseline cylinder and changes in cylinder, sphere or SER over 1 year, suggesting that baseline cylinder may not be useful in predicting refractive changes. However, it is important to consider the retrospective nature of this study, as well as the low level of baseline astigmatism (median −0.50 [interquartile range −0.75, 0.00] in the HAL group and median −0.25 [−0.50, 0.00] in the peripheral modification group). Further research is required to explore this effect in children with moderate and high levels of astigmatism at baseline. In agreement with Domsa et al.,^71^ this study suggests that optical interventions may influence astigmatism directly. However, it was also reported that the efficacy of the intervention in slowing spherical change was not influenced by astigmatism. Further research is required to explore the effects of optical interventions and astigmatism and how astigmatism may influence the efficacy of optical interventions.

A retrospective study in Chinese children over 12 months explored astigmatic changes in those wearing DIMS lenses (*n* = 179).^70^ Wang et al. reported that a larger myopic change in SER was associated with a greater change in astigmatism (change in astigmatism defined as >0.25D) (OR 1.73, 95% CI: 1.32–2.27). This relationship was dose‐dependent, with the OR increasing from 2.82 (1.06–7.49) for those with a change in SER of 0.25–0.75D to OR 6.32 (2.48–16.07) in those with >0.75D change in SER. Conversely, the change in AL was not associated with a shift in astigmatism. The authors also reported that baseline corneal astigmatism was associated with the change in astigmatism (OR = 1.17, 1–1.36). However, because there was no control group, the interaction between myopia control spectacles and change in astigmatism remains unknown. There was also a low threshold for the change in astigmatism (0.25D), which may have overestimated the findings.

Differences in the efficacy of toric versus spherical orthokeratology in children and adolescents with astigmatism was explored by Hong et al.^79^ The authors found that those wearing toric orthokeratology lenses achieved greater slowing of progression after a 1‐year period (0.20 mm vs. 0.47 mm, respectively). However, data were limited to 1 year of follow‐up and not all confounding factors were accounted for. In particular, data regarding parental myopia were not collected. The finding suggests that astigmatism may be implicated in the effectiveness of myopia control interventions. In agreement, a novel study using multifocal rigid gas permeable lenses identified corneal curvature as a significant predictor of axial elongation in a multivariate analysis model, which was significantly negatively correlated with axial elongation (*r* = −0.51).^69^

### Aim 2: Negative findings relating to myopia control and astigmatism

Zhang and Chen^66^ (*n* = 62) reported that the rate of axial elongation was not associated with the initial corneal astigmatism, where axial elongation was the outcome. This agrees with Chen et al.^76^ (*n* = 80) where no significant correlation was found between the rate of axial elongation and baseline astigmatism in Chinese children wearing either toric orthokeratology lenses or single vision spectacles. A retrospective study of clinical records of children in China (*n* = 249) undergoing orthokeratology also reported no significant correlation between baseline astigmatism and the rate of axial elongation.^65^ This is again in agreement with a recent 1‐year retrospective study (*n* = 542 eyes from 300 participants) in Chinese children.^68^

When comparing the effects of toric versus spherical orthokeratology (*n* = 50), Jiang et al.^58^ reported no significant difference in AL changes between those wearing toric (0.13 mm ± 0.18 mm) versus spherical lenses (0.11 mm ± 0.20), in a small case control study (*n* = 25). This indicates that full correction of astigmatism does not affect the effectiveness of orthokeratology in slowing myopia progression.

In contrast, Zhang and Chen^66^ reported that axial elongation was significantly less in the toric (0.04 ± 0.13 mm) than in the spherical orthokeratology group (0.09 ± 0.13 mm). However, the mean difference was small and may fall within the variability of the instrumentation used. These conflicting findings may be due to differences in the study protocol whereby Jiang et al.^58^ recruited children with astigmatism up to 1.50D, whereas Zhang and Chen^66^ recruited children with 1.50 to 3.50D astigmatism.

A number of investigations have also explored the effects of low dose (0.01%, 0.02%) atropine on corneal and refractive astigmatism in China.^74^, ^75^, ^77^, ^78^, ^83^ Wang et al.^74^, ^75^ investigated how the ocular biometrics of Chinese children changed following instillation of 0.01% or 0.02% atropine over a 2‐year period as part of a prospective cohort trial. No significant changes were observed in corneal curvature or corneal power. The Low Concentration Atropine for Myopia‐Progression (LAMP) study^77^ (*n* = 383) reported corneal astigmatism remained stable after 1 year, with no significant difference between children using 0.05% atropine versus those receiving 0.025% or 0.01% concentrations. In contrast, Zhu et al.^78^ noted a statistically significant, yet small, increase in cylindrical refractive error in 119 Chinese children receiving 0.01% atropine (−0.14 ± 0.29D), in comparison with the control group (−0.04 ± 0.23D). These findings may not be not clinically significant. Additionally, the study involved a small clinical sample (*n* = 94) with a number of participants lost to follow‐up during COVID: 14 and 13 in the treatment and control groups, respectively. The results were also confined to a single hospital‐based population. The Atropine for the Treatment of Myopia (ATOM) study (*n* = 400)^83^ also reported a small, statistically significant increase in corneal (J_0_) astigmatism in the 1% atropine group (0.15 ± 0.22) in comparison with the control group (0.07 ± 0.16D). However, these findings again came from a very small sample, may lie within the repeatability of instrumentation and are unlikely to be clinically meaningful.

Interestingly, Gao et al.^39^ explored whether a much higher concentration of atropine (1%), used twice a day for 5 days, affected the ocular components of Chinese children (*n* = 135). They reported that although there were small decreases in corneal power in the myopic eyes versus the hyperopic eyes at 3, 5 and 7 mm eccentricity, these changes were again small (<0.25D) and unlikely to be clinically meaningful. This study is limited by its short duration and the high concentration of atropine, which is used less commonly in clinic populations for myopia control.

## DISCUSSION

This literature review identified 47 studies which explored myopia, its progression and astigmatism. There were considerably more investigations exploring the association between myopia, its progression and astigmatism (*n* = 30) than those evaluating myopia control and astigmatism (*n* = 17). The majority of this research has been conducted in Asia (*n* = 31), in children only (*n* = 39), and the most common types of studies have either been longitudinal observational (*n* = 13) or retrospective in design (*n* = 14). The use of retrospective studies may increase the risk of bias and limit any conclusions that can be drawn about the causative role of astigmatism in myopia or vice versa^86^ Further prospective research in non‐Asian populations is required, particularly on the topic of myopia control interventions and astigmatism.

A variety of study designs have been used, including different methods of measuring astigmatism with varying inclusion and exclusion criteria. Additionally, there were differences in how myopia was defined across the studies, including the use of spherical power versus SER or differing thresholds for high myopia. All these factors impede the direct comparability of study findings and make it difficult to determine if astigmatism actively influences or is a consequence of myopic eye growth.

These disparities may have contributed to the conflicting results as to whether the presence of astigmatism is associated with the rate of myopia progression. Many of the studies exploring this topic have reported associations between astigmatism and myopia progression, with only a few observing an independent relationship between astigmatism in myopia progression^42^, ^50^ and none finding a causative role of astigmatism in promoting myopia progression. Further research is required to explore if astigmatism does indeed play a causative role in myopia progression (or vice versa) or if astigmatism is simply a biomarker of myopic eye growth.

Research indicates that astigmatism in infancy and early childhood may precede myopia onset and progression in later years.^47^, ^61^, ^62^ This suggests that infants and younger children with astigmatism should be monitored due to the risk of myopia in the future. However, findings do not provide clarity as to whether astigmatism causes myopia directly, or if astigmatic children become myopic because of shared environmental and lifestyle risk factors. Interestingly, recent research^19^ following COVID lockdowns has found that the prevalence and severity of astigmatism increased in response to the significant change in lifestyle, likely with more time indoors and spent on near activities such as viewing screens.^87^ These findings suggest that astigmatism may be influenced by similar lifestyle factors as myopia. However, a causal role for astigmatism in myopia progression is not yet proven.

This scoping review also included studies which reported if myopia was associated with increasing astigmatism. Some results indicated that greater astigmatic progression was associated with myopia,^50^, ^55^, ^84^ although other studies found no difference between those with stable or changing astigmatism.^48^ However, research is conflicting as to whether the prevalence of myopia is associated with the magnitude of astigmatism. Some have found that those with myopia are more likely to have astigmatism and that the prevalence of astigmatism increases with increasing myopia (*n* = 10 studies). For example, Tong et al. reported that those with myopia have a greater incidence of astigmatism.^55^ Other investigations^60^, ^63^, ^64^, ^73^, ^80^, ^82^ do not support this association (six studies) and instead either reported no association between myopia and astigmatism, or alternatively that astigmatism is associated with increasing levels of both myopia and hyperopia. This would suggest that astigmatism is implicated in both of these refractive errors. However, the lack of agreement between studies impairs any definitive conclusions and it remains unclear if astigmatism is simply a result of myopia or that it influences myopic eye growth.

Research has explored whether the type of astigmatism may be associated with myopia and its progression. Studies in adults have reported that high myopia may be associated with higher levels of WTR astigmatism.^44^, ^62^ However, some investigations in children have reported that ATR astigmatism was associated with myopia and its progression,^46^, ^47^, ^49^ while others have either observed the opposite finding^56^ or no association between the type of astigmatism and myopia.^59^, ^60^ Thus, the type of astigmatism is not consistently associated with myopia and perhaps does not influence myopic eye growth.

A total of 13 investigations were explored which evaluated myopia control strategies and astigmatism, which were largely confined to studies of orthokeratology and atropine. It was found that corneal power and curvature do not change following the instillation of topical atropine. The effects of uncorrected astigmatism on the rate of myopia progression when using other forms of myopia management are unknown. A few studies explored differences in the efficacy of toric versus spherical orthokeratology lenses, which may indirectly evaluate the effects of uncorrected astigmatism on myopia. However, results are conflicting as to whether full astigmatic correction is required for maximum myopia control. Research may be limited on this topic due to ethical concerns surrounding the undercorrection of refractive error and the resulting effects on vision. Uncorrected astigmatism cannot be corrected by accommodation, and the degradation of image quality that occurs even with small levels of astigmatism^88^ may be sufficient to disrupt emmetropisation. This has been evidenced in animal models where small amounts of form deprivation myopia can disrupt eye growth and stimulate myopia.^89^

Although the implications of uncorrected astigmatism in humans are unclear, the retrospective study by Xu et al.^67^ indicated that upon analysis of hospital records, there may be an interaction between myopia and astigmatism. This recent study reported an acceleration in cylindrical refraction due to a reduction in the progression of the sphere while wearing myopia control spectacles. The authors concluded that the change in astigmatism was due to the direct effects of wearing myopia control spectacles and indirectly due to the slowing of spherical changes. However, it is unclear whether the control group included stable or progressing myopes. Although matched by age and SER, these children did not select intervention, which may suggest that their myopia was already stable. This study highlights that further research is required to understand better the mechanism underpinning the effects of these specialised spectacle lenses for myopia control.

Further research is also required to explore if the effectiveness of optical‐based interventions varies with the level of uncorrected or corrected astigmatism. This is of particular interest as current myopia control spectacle lenses are available to correct a range of astigmatism. A recent study has explored the effectiveness of the DIMS spectacle lens in a clinical population exhibiting a range of refractive errors.^71^ However, data were neither analysed by level of astigmatism, nor did the authors report the astigmatic characteristics of the sample. Interestingly, the study found that in those wearing DIMS spectacles, participants with astigmatism demonstrated greater myopia progression. However, this finding was not reported in children wearing single vision lenses. This suggests that the presence of astigmatism may be detrimental to the effectiveness of optical interventions. The authors proposed two possible theories. One is that astigmatism may affect the uniformity of the peripheral blur induced by the DIMS lenses, thereby reducing the effectiveness of the intervention. Alternatively, changes in axial elongation and astigmatism may be interlinked and directly influenced by optical interventions such as the DIMS lens. This is similar to the findings of Xu et al.,^67^ where greater astigmatic progression occurred in participants wearing HAL lenses. The authors suggested that HAL lenses may directly accelerate astigmatic progression. They also propose an indirect effect on astigmatism, whereby the slowing in spherical progression may promote an increase in astigmatism. Again, this suggests an interdependent relationship between spherical and astigmatic changes. Further research is required to explore these theories and to understand better the impact of astigmatism on the effectiveness of myopia interventions, as well as the effects of such interventions on astigmatism. Although Xu et al.^67^ explored whether baseline astigmatism was associated with changes in sphere and SER in those wearing myopia control spectacles, the baseline level of astigmatism was low. Therefore, it remains unclear whether moderate or high levels of astigmatism affect the effectiveness of such interventions. Despite the limitations of these two studies, they have indicated that myopia control interventions may both influence and be influenced by the presence of astigmatism. The range of astigmatism included in this scoping review is likely to be representative of the overall population, with 28 studies being population‐based and not including an upper limit on astigmatism (Table 2). One limitation of the research is that a number of studies did not analyse the spherical component separately but instead explored the association between cylinder and SER. By solely looking at the change in SER rather than sphere and cylinder individually, the true effects of astigmatism and myopia may be masked.

The prevalence of moderate (−0.75DC to −1.50DC) and high astigmatism (≤ −3.00DC) in children aged 7–19 years has been reported to be, on average, 9.3% and 1.8%, respectively.^90^ Research on the prevalence of moderate and high astigmatism in children is limited and usually includes adult populations, which may skew the prevalence of astigmatism since it generally increases with age in adulthood.^11^ Despite the lack of research on prevalence, these numbers suggest that children with moderate and high astigmatism are likely to contribute to the patient base seen by clinicians prescribing myopia control therapies. Further research is required to determine the effectiveness of myopia control interventions in those with greater astigmatism. This is necessary to develop an evidence base for clinical decision making and patient management plans in myopic children with astigmatism.

It is important to recognise the limitations of this scoping review, which did not formally appraise the quality of the evidence using a standardised assessment tool (e.g., GRADE), typically included in systematic reviews. However, the aim of this review was to identify knowledge gaps and refine research questions, acting as a precursor to a systematic review. The findings indicate that the relationship between astigmatism and myopia requires further formal analysis in the form of systematic and meta‐analysis.

## CONCLUSION

Astigmatism in the younger years may indicate a greater likelihood for myopia onset and progression. The prevalence of astigmatism also seems to increase with the level of myopia. However, research on the role of the type of astigmatism in myopia development and progression is conflicting. Many studies reported associations, but few explored a causative role of astigmatism in promoting myopia, making it unclear if astigmatism is simply a biomarker of myopic eye growth. The diverse range of study designs and methodologies employed may also impair the comparability of study findings. Further research is required in non‐Asian populations to determine if results are comparable with other ethnicities/regions. Research on the role of astigmatism, both corrected and uncorrected, on the effectiveness of myopia control interventions is needed, including determination of the efficacy of such interventions in children with moderate and high levels of astigmatism. This information is needed to provide an evidence base to inform clinical decision‐making and patient management plans for myopic children with astigmatism. Clinicians should be cautious in discussing the likelihood of success using optical interventions in patients with astigmatism until an evidence base has been established.

## AUTHOR CONTRIBUTIONS

Stephanie Kearney: Data curation (lead), formal analysis (lead), methodology (lead), validation (lead), visualisation (lead), writing—original draft (lead) and writing—review and editing (lead). Rakhee Shah: Conceptualisation (lead), data curation (supporting), formal analysis (supporting), methodology (supporting), validation (supporting), visualisation (supporting), writing—original draft (supporting) and writing—review and editing (supporting). Natalia Vlasak: Conceptualisation (lead) and writing—review and editing (supporting).

## FUNDING INFORMATION

None.

## CONFLICT OF INTEREST STATEMENT

NA is an employee of HOYA Vision Care. RS is a previous employee of HOYA Vision Care. SK has received consultancy and lecturing fees from HOYA Vision Care.
